# Association of maternal IL-2, IL-4, and IL-13 with intrauterine growth restriction and umbilical artery doppler: a case-control study

**DOI:** 10.61622/rbgo/2026rbgo22

**Published:** 2026-03-20

**Authors:** Bahareh Mousavi Afshar, Roghayeh Dargahi, Shervin Tabrizian, Maryam Jafarzadeh, Sajad Sarkhani, Nasrin Sarkhani

**Affiliations:** 1 Ardabil University of Medical Sciences School of Medicine Department of Gynecology and Obstetric Ardabil Iran Department of Gynecology and Obstetric, School of Medicine, Ardabil University of Medical Sciences, Ardabil, Iran; 2 Azad University of Medical Sciences School of Medicine Tehran Iran School of Medicine, Azad University of Medical Sciences, Tehran, Iran; 3 Ardabil University of Medical Sciences Fatemi Hospital Department of Pathology Ardabil Iran Department of Pathology, Fatemi Hospital, Ardabil University of Medical Sciences, Ardabil, Iran

**Keywords:** Pregnancy, Fetal growth retardation, Umbilical arteries, Ultrasonography, doppler, Ultrasonography, prenatal, Prenatal diagnosis, Interleukin-2, Interleukin-4, Interleukin-13

## Abstract

**Objective:**

Maternal pro-inflammatory responses have been implicated in pregnancy complications. This study examined the association of maternal serum IL-2, IL-4, and IL-13 levels with intrauterine growth restriction (IUGR) and umbilical artery Doppler indices.

**Methods:**

This case-control study was conducted at Alavi Teaching and Referral Hospital, Ardabil, Iran, from August 2024 to June 2025. Sixty singleton pregnant women (30 with IUGR and 30 with normal pregnancies) between 28–36 weeks of gestation were included. Umbilical artery resistive (RI) and pulsatility (PI) indices were assessed by a single sonographer, and serum cytokine levels were measured using ELISA. Statistical analyses (t-test, chi-square, Pearson's correlation, logistic regression) were performed with SPSS v25. The study was approved by the Ethics Committee of Ardabil University of Medical Sciences (no. 1403.049).

**Results:**

Compared with controls, the IUGR group had higher IL-2 and lower IL-4 and IL-13 levels, as well as increased PI and RI (all P<0.05). Elevated IL-2 (OR=1.027; 95%CI: 1.011–1.044; P=0.001) and reduced IL-4 (OR=0.963; 95%CI: 0.929–0.999; P=0.042) and IL-13 (OR=0.978; 95%CI: 0.962–0.995; P=0.009) were independently associated with IUGR. IL-2 correlated positively, while IL-4 and IL-13 correlated negatively, with both Doppler indices (all P<0.05).

**Conclusions:**

Pregnant women with IUGR exhibited a pro-inflammatory Th1-biased cytokine profile, characterized by increased IL-2 and decreased IL-4 and IL-13, which correlated with elevated umbilical vascular resistance. These findings suggest an immunologic contribution to placental dysfunction. However, given the observational design and small sample size, causal inferences cannot be made, and confirmation in larger multicenter studies is warranted.

## Introduction

Intrauterine growth restriction (IUGR) affects approximately 10 to 15% of pregnancies, predominantly in developing countries.^(1)^ IUGR increases the risk of perinatal complications, including birth asphyxia, persistent pulmonary hypertension, metabolic disturbances, feeding difficulties, necrotizing enterocolitis, sepsis, and long-term neurodevelopmental impairments.^(1,2)^

Successful pregnancy requires fine regulation of maternal immune responses that promote fetal tolerance while preserving protective immunity.^(3)^ Dysregulation of this balance, particularly through excessive pro-inflammatory activity, has been implicated in adverse pregnancy outcomes including preeclampsia, miscarriage, and preterm birth.^(4-7)^ However, the contribution of specific immunoregulatory cytokines to IUGR remains insufficiently characterized.^(8,9)^

Interleukin-2 (IL-2) is a Th1-type cytokine that promotes T-cell proliferation and natural killer (NK) cell activation and has been associated with pathological inflammation in pregnancy disorders such as preeclampsia and preterm birth.^(10-14)^ In contrast, interleukin-4 (IL-4) and interleukin-13 (IL-13) are Th2-type cytokines that downregulate pro-inflammatory mediators and contribute to maternal–fetal tolerance at the placental interface.^(15-17)^ Reduced IL-4 and IL-13 levels have been observed in pregnancies complicated by IUGR and other immune-mediated disorders.^(8,9,18-20)^

Collectively, these findings suggest that an imbalance between pro- and anti-inflammatory cytokines may play a role in IUGR pathogenesis. Nevertheless, data on IL-2, IL-4, and IL-13 patterns in maternal circulation and their correlation with fetoplacental hemodynamics remain limited. This study therefore aimed to investigate the association of maternal serum IL-2, IL-4, and IL-13 levels with IUGR and umbilical artery Doppler indices.

## Methods

This analytical observational case–control study was conducted at Alavi Teaching and Referral Hospital, Ardabil, Iran, from August 1, 2024, to June 30, 2025. The study population comprised pregnant women who attended the hospital during the study period. Cases were consecutive patients diagnosed with IUGR based on clinical criteria and Doppler ultrasound findings. Controls were women with uncomplicated pregnancies receiving routine antenatal care at the same hospital. Controls were recruited contemporaneously using frequency matching to achieve comparable distributions of maternal age, body mass index (BMI), gravidity, parity, and gestational age.

Inclusion criteria were singleton pregnancy, maternal age <40 years, and gestational age between 28 and 36 weeks. Exclusion criteria comprised other pregnancy complications; chronic systemic diseases (e.g., pregestational diabetes, cardiovascular or renal disease, anemia); history of IUGR in previous pregnancies; conception via assisted reproductive technologies; early termination of pregnancy; fetal congenital anomalies; substance abuse, tobacco smoking, or teratogenic drug use; inability to measure cytokine levels; and participant withdrawal.

No formal a priori sample-size calculation was performed due to lack of prior comparable data; 30 cases and 30 controls were enrolled based on feasibility and recruitment capacity. For transparency, post-hoc observed power for key predictors in the logistic regression was calculated (see Limitations).

At enrollment, demographic and clinical data were recorded. Umbilical artery Doppler ultrasonography was performed using a GE Voluson E10 system by a single experienced sonographer to minimize inter-operator variability. Measurements were obtained with participants in the supine position following a short rest period. Doppler sampling was performed on the free loop of the umbilical cord with the insonation angle ≤30°, and three similar consecutive waveforms were recorded; the mean values of the pulsatility index (PI) and resistive index (RI) were used for analysis. The sonographer was blinded to cytokine assay results. Venous blood samples were collected at the time of Doppler assessment. Serum was separated and stored at −80°C until analysis. Concentrations of IL-2, IL-4, and IL-13 were measured using validated commercial ELISA kits following the manufacturers’ protocols. Laboratory personnel were blinded to clinical group allocation. Participants were followed throughout pregnancy to monitor obstetric care and fetal well-being. Decisions regarding timing and mode of delivery were made based on clinical indications and ultrasound findings.

Normality of continuous variables was assessed using the Shapiro–Wilk test. For group comparisons, the independent-samples t-test or Mann–Whitney U test was used as appropriate. Categorical variables were compared using the Chi-square test. Correlations between cytokines and Doppler indices were assessed using Spearman's rho. Multivariable logistic regression was performed to estimate associations between cytokine levels and odds of IUGR, adjusting for maternal age, gestational age, gravidity, parity, and BMI. A two-sided P < 0.05 indicated statistical significance.

The study protocol complied with the ethical standards of the Declaration of Helsinki and received approval from the Research Ethics Committee of Ardabil University of Medical Sciences (no. 1403.049). Written informed consent was obtained from all participants before enrollment.

## Results

A total of 60 pregnant women (30 IUGR, 30 controls) were included. Baseline maternal and obstetric characteristics did not differ significantly between groups. Compared with the control group, the IUGR group exhibited significantly higher serum IL-2 levels (*P* = 0.013), whereas IL-4 (*P* = 0.002) and IL-13 (*P* < 0.001) levels were significantly lower. Moreover, Doppler indices were markedly elevated in the IUGR group (*P* < 0.001) (Table 1).

**Table 1:** Demographic and obstetric characteristics, circulating interleukin levels, and Doppler indices among study participants

Variables		Groupe		p-value
		IUGR	Control	
Maternal age (years)		31.0 ± 6.2 31.0 (25.8 – 35.0)	28.6 ± 5.0 27.0 (25.0 – 33.3)	0.100^†^
Gestational age (weeks)		32.3 ± 2.4 32.5 (30.0 – 35.0)	31.5 ± 2.3 31.5 (29.8 – 33.3)	0.172^‡^
Gravidity				
	1–2	24 (80.0)	25 (83.3)	0.739^§^
	3–5	6 (20.0)	5 (16.7)	
Parity				
	nulliparous	18 (60.0)	16 (53.3)	0.602^§^
	multiparous	12 (40.0)	14 (46.7)	
BMI (kg/m²)		29.1 ± 5.4 28.0 (24.4 – 33.8)	28.0 ± 4.3 29.2 (24.2 – 31.2)	0.391^†^
IL-2 (pg/mL)		445.3 ± 186.7 409.4 (320.5 – 585.5)	342.6 ± 155.0 291.3 (240.0 – 391.0)	0.013^‡^
IL-4 (pg/mL)		126.2 ± 75.8 101.2 (79.4 – 155.7)	176.5 ± 86.2 146.1 (117.9 – 189.0)	0.002^‡^
IL-13 (pg/mL)		107.7 ± 111.1 60.5 (46.5 – 101.3)	241.5 ± 125.1 208.7 (167.6 – 242.2)	< 0.001^‡^
PI		1.56 ± 0.2 1.53 (1.38 – 1.70)	0.94 ± 0.08 0.95 (0.87 – 1.01)	< 0.001^‡^
RI		0.82 ± 0.04 0.83 (0.79 – 0.85)	0.51 ± 0.04 0.51 (0.47 – 0.54)	< 0.001^‡^

Data are presented as mean ± standard deviation (SD) and median (IQR) for continuous variables, and as frequency (percentage) for categorical variables; P-values were calculated using the independent-samples t-test (†), Mann–Whitney U test (‡), or Chi-square test (§), as appropriate; BMI: body mass index; IL-2: interleukin-2; IL-4: interleukin-4; IL-13: interleukin-13; PI: pulsatility index; RI: resistive index

In multivariable logistic regression, higher IL-2 was associated with increased odds of IUGR (OR = 1.027; 95% CI: 1.011–1.044; P = 0.001), while higher IL-4 (OR = 0.963; 95% CI: 0.929–0.999; P = 0.042) and IL-13 (OR = 0.978; 95% CI: 0.962–0.995; P = 0.009) were associated with lower odds of IUGR (Table 2).

**Table 2:** Logistic regression analysis of factors associated with the risk of IUGR

Variables	B	Standard error	p-value	Odds ratio	95% C.I. for EXP(B)	
					Lower	Upper
Maternal age	0.001	0.102	0.998	1.000	0.818	1.223
Gestational age	0.155	0.248	0.533	1.167	0.718	1.899
Gravidity	- 0.437	0.724	0.546	0.646	0.156	2.671
Parity	- 0.577	1.498	0.700	0.562	0.030	10.574
BMI	0.114	0.143	0.423	1.121	0.847	1.483
IL-2	0.027	0.008	0.001	1.027	1.011	1.044
IL-4	- 0.037	0.018	0.042	0.963	0.929	0.999
IL-13	- 0.022	0.008	0.009	0.978	0.962	0.995

B: regression coefficient (Wald); 95% C.I. for Exp(B): 95% confidence interval for the odds ratio; BMI: body mass index; IL-2: interleukin-2; IL-4: interleukin-4; IL-13: interleukin-13

Spearman's correlation analysis revealed that the PI was positively correlated with IL-2 (*r* = 0.390, *P* = 0.002) and negatively correlated with IL-4 (*r* = −0.364, *P* = 0.004) and IL-13 (*r* = −0.508, *P* < 0.001). Similarly, the RI was positively correlated with IL-2 (*r* = 0.301, *P* = 0.019) and negatively correlated with IL-4 (*r* = −0.254, *P* = 0.050) and IL-13 (*r* = −0.472, *P* < 0.001).

## Discussion

In this case-control study of 30 IUGR pregnancies and 30 matched controls, we observed a distinct cytokine profile in the IUGR group. Maternal serum IL-2 levels were significantly higher in IUGR, whereas IL-4 and IL-13 levels were significantly lower. In multivariable analysis, higher IL-2 was associated with increased odds of IUGR, while higher IL-4 or IL-13 were associated with decreased odds. Additionally, umbilical artery Doppler indices (PI and RI) were positively correlated with IL-2 and inversely correlated with IL-4 and IL-13.

Our observation of elevated IL-2 and reduced IL-4/IL-13 in IUGR aligns with a Th1-dominant, pro-inflammatory shift. IL-2 acts as a Th1 cytokine,^(10)^ whereas IL-4 and IL-13 mediate Th2 and anti-inflammatory responses.^(15,21)^ Similar patterns have been described previously: Al-Azemi et al.^(8)^ found lower IL-4 in IUGR compared with normal pregnancies, and Raghupathy et al.^(9)^ observed reduced IL-13 production. Together, these findings indicate Th2 suppression in IUGR. In contrast, pro-inflammatory cytokines tend to be elevated: Raghupathy et al.^(9)^ observed higher IL-8 and other Th1 cytokines in IUGR. Although we were unable to identify prior reports of maternal serum IL-2 concentrations in IUGR, IL-2's role as a Th1 mediator supports its elevation in an inflammatory state. Overall, these patterns support the concept that IUGR is associated with increased maternal Th1 (inflammatory) activity and reduced Th2 (anti-inflammatory) signaling.

We found that higher umbilical artery PI and RI paralleled the cytokine imbalance: IL-2 correlated positively with PI/RI, whereas IL-4 and IL-13 correlated negatively. Few studies have directly examined such correlations. One investigation in preeclamptic women reported that serum IL-4 correlated positively with umbilical artery PI.^(22)^ Although that study focused on preeclampsia rather than IUGR, it similarly suggests a link between inflammatory signals and Doppler flow. To our knowledge, no published data exist on IL-2 or IL-13 versus Doppler indices in IUGR.

The direction of our findings appears physiologically plausible, as pregnancies with higher placental resistance tended to exhibit elevated pro-inflammatory cytokines along with reduced anti-inflammatory cytokines. For instance, lower IL-4, an anti-inflammatory cytokine, was associated with higher PI, suggesting that impaired placental perfusion occurs alongside suppressed Th2 activity. Taken together, these results point to a coherent biological picture in which IUGR pregnancies exhibit a Th1-skewed immune profile linked to abnormal fetoplacental blood flow. Elevated IL-2 and reduced IL-4/IL-13 may contribute to placental inflammation and dysfunction, reflected by higher umbilical artery Doppler indices. Conversely, a balanced or Th2-dominant cytokine profile may support better (lower-resistance) placental perfusion. The parallel patterns in immune and Doppler findings support an association between maternal immune dysregulation and IUGR pathophysiology. However, this relationship is observational, and causality cannot be inferred. It is also possible that placental insufficiency triggers systemic inflammation, rather than results from it.

From a clinical perspective, our findings highlight the potential value of maternal cytokine profiling as an adjunct tool for early identification of pregnancies at risk of IUGR. Elevated IL-2 and reduced IL-4/IL-13 levels may reflect an underlying inflammatory–immune imbalance that precedes detectable Doppler abnormalities. Growing evidence supports the potential of maternal cytokine profiles as early, non-invasive biomarkers to identify pregnancies at risk for placental disorders,^(23-25)^ although clinical implementation requires validation in larger, prospective cohorts. Future research should assess whether longitudinal cytokine monitoring can aid in differentiating IUGR from other inflammatory pregnancy complications such as preeclampsia and improve timing of intervention.

This study has several limitations that should be considered when interpreting the findings. First, the modest sample size (30 cases and 30 controls), chosen based on feasibility rather than a formal a priori power calculation, increases the risk of type II error and limits precision. For transparency we conducted post-hoc power calculations based on the logistic regression Wald statistics: observed power was approximately 92% for IL-2, 79% for IL-13, and 54% for IL-4. These results indicate that the study had adequate power to detect the observed IL-2 association, moderate power for IL-13, but limited power for IL-4; accordingly, findings regarding IL-4 should be interpreted with caution and merit confirmation in larger cohorts. Reporting of effect sizes (ORs) and 95% CIs is provided to allow readers to assess estimate precision. Second, the case–control design and non-random sampling of controls may introduce selection bias despite the use of frequency matching for key maternal characteristics. Residual confounding remains possible. Third, we measured cytokines and performed Doppler assessment at a single mid- to late-gestation time point, which does not allow evaluation of longitudinal or dynamic changes in cytokine patterns throughout pregnancy. Moreover, we measured maternal serum cytokines rather than placental or fetal compartments, so the results reflect maternal systemic inflammation, not necessarily local placental cytokine expression. Fourth, exclusion of women with chronic systemic diseases, multiple gestation, or other pregnancy complications strengthens internal validity but restricts the external applicability of the results to broader obstetric populations. Fifth, our findings are specific to the population studied (a single center in Iran) and to singleton pregnancies with gestational ages of 28–36 weeks. The results may not apply to very early-onset IUGR, multiple pregnancies, or populations with different demographics. These factors limit generalizability to other populations and clinical contexts.

## Conclusion

This study demonstrated that pregnancies complicated by IUGR are characterized by a distinct maternal cytokine profile, with elevated IL-2 and reduced IL-4/IL-13 levels associated with increased umbilical artery Doppler indices. These findings support a Th1-skewed inflammatory milieu potentially linked to placental vascular dysfunction. While the observational design precludes causal inference, the results highlight the potential clinical relevance of maternal cytokine assessment in understanding and possibly identifying IUGR risk. Future large, multicenter studies are warranted to validate these associations and to explore whether cytokine profiles could serve as early biomarkers or therapeutic targets in placental disorders.

## Data Availability

The research data are described in the article presented.
